# DETECTING PRE-FRAILTY STATUS: COMPARISON OF CLINICAL JUDGMENTS AND THE PAULSON LICHTENBERG FRAILTY INDEX.

**DOI:** 10.1093/geroni/igz038.3280

**Published:** 2019-11-08

**Authors:** Yi-Ling Hu, Heather A Fritz

**Affiliations:** Wayne State University, Detroit, Michigan, United States

## Abstract

Nearly 50% of U.S. elders are prefrail and at risk for frailty. Identifying prefrail elders and escalating care could attenuate frailty progression. Screening tools are seldom used in practice. Thus, clinical judgment may be a realistic way to ensure widespread frailty screening. No studies, however, have assessed the validity of clinicians’ judgment in identifying prefrail elders. This study explored the level of agreement between clinical judgments of frailty status and status categorizations made using the validated Paulson Lichtenberg Frailty Index (PLFI). Older Blacks (n = 202) recruited from a primary care clinic were first categorized as healthy, pre-frail, or frail using the PLFI. Next, geriatric physicians and nurses categorized participants into one of the same categories based on clinical judgment. Clinicians could use medical records to make determinations. We used Cohen’s Kappa to determine the level of agreement of both approaches. We used descriptive statistics to explore if any of the 5 PLFI indicators explained discordant categorizations. Of the 202 participants (mean age: 76.7 8.6), 52 (26%) were prefrail and 57 (28%) were frail based on the PLFI. Physicians’ judgments aligned with the PLFI in 43% of prefrail and 65.7% of frail cases. Nurse judgments aligned with the PLFI in 43.9% of prefrail and 17% of frail cases. There was slight to fair agreement between clinical judgments and PLFI (physicians Cohen’s κ = .23; Nurses Cohen’s κ = .59). No specific PLFI indicators independently explained discordant categorizations. Findings suggest that clinical judgments did not align well with PLFI categorizations.

